# Nicotinamide Riboside Supplementation for Treating Elevated Systolic Blood Pressure and Arterial Stiffness in Midlife and Older Adults

**DOI:** 10.3389/fcvm.2022.881703

**Published:** 2022-05-10

**Authors:** Kaitlin A. Freeberg, Daniel H. Craighead, Christopher R. Martens, Zhiying You, Michel Chonchol, Douglas R. Seals

**Affiliations:** ^1^Integrative Physiology of Aging Laboratory, Department of Integrative Physiology, University of Colorado, Boulder, Boulder, CO, United States; ^2^Department of Kinesiology and Applied Physiology, University of Delaware, Newark, DE, United States; ^3^Division of Renal Diseases and Hypertension, University of Colorado Anschutz Medical Campus, Aurora, CO, United States

**Keywords:** aging, pulse wave velocity, NR, NMN, NAD^+^

## Abstract

**Background:**

Aging is the primary risk factor for cardiovascular diseases, the leading cause of death worldwide. Age-related increases in systolic blood pressure (SBP) link advancing age to cardiovascular disease risk. A key mechanism mediating the increase in SBP with aging is stiffening of the large elastic arteries, which occurs due to increases in oxidative stress, inflammation, and vascular smooth muscle tone. Nicotinamide adenine dinucleotide (NAD^+^) is a key molecule in energy metabolism and cellular functioning which declines with advancing age and chronic disease. Dietary supplementation with NAD^+^ precursors, such as nicotinamide riboside, boosts NAD^+^ bioavailability and may improve cardiovascular health. Here, we present the protocol for a randomized, controlled trial investigating the efficacy of 3 months of oral supplementation with nicotinamide riboside for decreasing SBP and arterial stiffness in midlife and older adults with initial above-normal (120–159 mmHg) SBP (ClinicalTrials.gov Identifier: NCT03821623). The primary outcome is casual (resting) SBP and secondary outcomes include 24-h SBP and aortic stiffness. Other outcomes include assessment of safety; tolerability; adherence; diastolic BP; systemic NAD^+^ bioavailability; and circulating biomarkers of oxidative stress, inflammation, and sympathoadrenal activity.

**Methods:**

A randomized, double-blind, placebo-controlled, single-site parallel-group design clinical trial will be conducted in 94 (47/group) midlife and older (age ≥ 50 years) adults with initial above-normal SBP. Participants will complete baseline testing and then will be randomized to either nicotinamide riboside (500 mg, 2×/day, NIAGEN^®^; ChromaDex Inc.) or placebo supplementation. Outcome measures will be assessed again after 3 months of treatment.

**Discussion:**

This study is designed to establish the safety and efficacy of the NAD^+^ boosting compound, nicotinamide riboside, for reducing casual and 24-h SBP and aortic stiffness in midlife and older adults with above-normal SBP at baseline, a population at increased risk of cardiovascular diseases.

**Clinical Trial Registration:**

[www.ClinicalTrials.gov], identifier [NCT03821623].

## Introduction

### Background and Rationale

Aging is the primary risk factor for the development of cardiovascular diseases (CVD), the leading cause of death worldwide, as well as other chronic conditions (1, 2). The increased risk of CVD with advancing age is largely due to adverse changes to the vasculature, such as stiffening of the large elastic (i.e., aorta and carotid) arteries and resultant increases in systolic blood pressure (SBP) (3, 4). Arterial stiffening is mediated by changes to both the structural and functional components of the arterial wall induced by oxidative stress, inflammation, and sympathetic activation (5–9). It is estimated that only ∼30% of adults aged 50–59 years have healthy levels of arterial stiffness and BP and that the prevalence of so-called “healthy vascular aging” drops to only 1% in adults aged ≥ 70 years (10). Thus, establishing safe and efficacious strategies to lower SBP and improve vascular function is a biomedical necessity for reducing age-related CVD burden.

**FIGURE 1:**
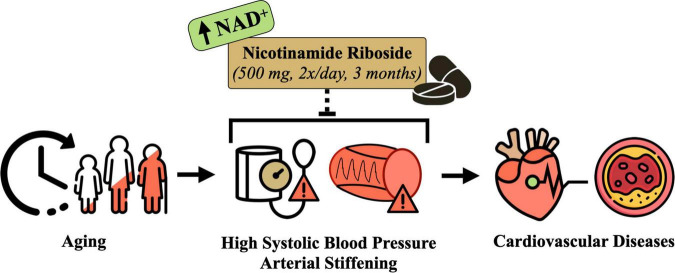
Conceptual overview of the hypothesized effects of nicotinamide riboside supplementation for treating elevated systolic blood pressure and arterial stiffness in midlife and older adults. Designed with resources from flaticon.com.

Advancing age is associated with declines in nicotinamide adenine dinucleotide (NAD^+^), a central coenzyme in energy metabolism and essential substrate for key enzymes involved in cellular functioning and homeostasis (11–15). Declines in NAD^+^ have been linked to multiple chronic diseases (16–18), such that boosting NAD^+^ bioavailability has emerged as a therapeutic strategy of interest for slowing or preventing age-related physiological dysfunction (14, 19, 20). Indeed, a number of on-going clinical trials are investigating the effects of NAD^+^-boosting compounds on key physiological outcomes that become impaired with aging, including glucose metabolism, insulin sensitivity, and skeletal muscle and cardiac function (17). However, there are limited published data or ongoing studies assessing the efficacy of increasing NAD^+^ bioavailability for improving BP and vascular function (Figure 1).

Our laboratory has utilized supplementation with the NAD^+^ precursors nicotinamide mononucleotide and nicotinamide riboside to restore NAD^+^ bioavailability (toward young levels) in preclinical and clinical studies, respectively (21, 22). These studies were conducted to provide initial evidence that these compounds are safe and efficacious for improving BP and/or vascular function in the context of healthy aging. We found that increasing NAD^+^ concentrations *via* dietary supplementation of nicotinamide mononucleotide lowered arterial stiffness in old mice (21), which provided initial proof-of-concept evidence that NAD^+^ precursors may be a beneficial intervention for CV aging. We then translated these findings into a pilot clinical study to gain preliminary insight into the safety and tolerability of nicotinamide riboside, a naturally occurring vitamin B_3_ derivative and commercially available dietary supplement (23), for improving NAD^+^ bioavailability in healthy midlife and older adults (22). We also leveraged this opportunity to assess the effects of nicotinamide riboside supplementation on physiological function to inform a subsequent larger clinical trial. Results from this crossover design pilot study suggested that 6 weeks of dietary supplementation with nicotinamide riboside was safe and well-tolerated, with no serious adverse events reported. Moreover, our data showed that nicotinamide riboside treatment effectively increased bioavailability of NAD^+^ and related NAD^+^ metabolites and lowered SBP (-4 mmHg), diastolic BP (DBP) (-2 mmHg), and arterial stiffness, assessed as carotid-femoral pulse wave velocity (CFPWV; –42 cm/s), in the overall group compared to placebo treatment. Importantly, *post hoc* exploratory subgroup analyses showed that the CV benefits of nicotinamide riboside supplementation may be greater in those with initial SBP in the above-normal (≥ 120 mmHg) vs. normal (< 120 mmHg) range, as SBP was reduced by 9 mmHg in the above-normal SBP subgroup. This reduction in SBP is clinically significant as it is associated with a 30–40% lower risk of CVD mortality (24). Thus, these encouraging preliminary results from our pilot clinical study led us to design the present protocol.

The current study (ClinicalTrials.gov Identifier: NCT03821623) is a phase IIa clinical trial investigating the efficacy of oral nicotinamide riboside supplementation for decreasing: (1) casual SBP; (2) 24-h SBP [a strong CVD risk factor independent of casual SBP (25–27)]; and (3) aortic stiffness in midlife and older adults with initial above-normal SBP (120–159 mmHg). We also will further assess the safety and tolerability of, and adherence to, the intervention as well as the potential underlying mechanisms that may mediate improvements in CV health with nicotinamide riboside treatment. To assess potential mechanisms of action, we will determine the effects of nicotinamide riboside supplementation on concentrations of NAD^+^; NAD^+^-related metabolites; and circulating markers of systemic oxidative stress, inflammation, and sympathoadrenal activity. We will address these aims by conducting a properly powered clinical trial with a clinically relevant treatment duration based on current BP treatment guidelines (3) investigating the effects of 3 months of nicotinamide riboside (500 mg, 2×/day) vs. placebo supplementation in midlife and older adults with above-normal SBP.

## Methods

### Study Design

This is a randomized, double-blind, placebo-controlled, parallel-group design, phase IIa clinical trial assessing the safety and efficacy of 3 months of nicotinamide riboside vs. placebo supplementation in 94 (47/group) midlife and older (age ≥ 50 years) adults with above-normal SBP at baseline. The duration of nicotinamide riboside supplementation for the current study was chosen based on clinical guidelines for the treatment of high BP, which recommend that patients with elevated BP or hypertension undergo non-pharmacological therapy, alone or in combination with BP-lowering medication, for at least 3 months before reevaluation of BP goals and medication regimens (3). This treatment duration also will extend upon findings from our previous crossover design pilot study in which subjects were administered nicotinamide riboside and placebo for 6-week periods (22). Midlife and older men and women with SBP 120–159 mmHg will be eligible to participate provided they meet all criteria for inclusion and exclusion (Table 1).

**TABLE 1:** Inclusion and exclusion criteria.

Inclusion criteria	Exclusion criteria
∙ Age ≥ 50 years: Women ≤55 years will be confirmed as postmenopausal based on cessation of menses for >1 year and, in women without a uterus, FSH > 40 IU/L.	• Other chronic medical condition (e.g., diabetes, chronic kidney disease, cancer)
• Ability to provide informed consent	• Postmenopausal < 1 year
• Willing to accept random assignment to a condition	• Current smoker
• Systolic blood pressure in the elevated to stage 2 hypertension range (120–159 mmHg)	• Uncontrolled thyroid disease or change in thyroid medication within previous 3 months
• Body mass index < 40 kg/m^2^	• Regular vigorous aerobic/endurance exercise (>4 bouts/week, >30 min/bout at a workload >6 METs)
• Weight stable in the prior 3 months (<2 kg change) and willing to remain weight stable throughout the study	• Blood donation within 8 weeks prior to enrolling in study; unwilling to abstain from donating blood for 8 weeks after completing the study
• Absence of other clinical disease as determined by medical history and blood chemistries: ° Total cholesterol < 240 mg/dL ° Fasting plasma glucose < 126 mg/dL	
• Mini-mental state examination score ≥ 21	
• Free from alcohol dependence or abuse, as defined by the American Psychiatry Association, Diagnostic and Statistical Manual of Mental Disorders (DSM-V)	

*FSH, follicle stimulating hormone; METs, metabolic equivalents.*

### Outcomes

All outcomes will be measured at baseline and after the 3-month supplementation period. All study outcomes (i.e., vascular measurements and blood sampling) will be collected after a > 5-h fast from food (water allowed) and caffeine, > 24-h abstention from alcohol and physical activity, and > 48-h abstention from dietary supplements and over the counter medications. Check-in visits will be performed without restrictions.

### Primary Outcome

#### Casual Systolic Blood Pressure

**FIGURE 2:**
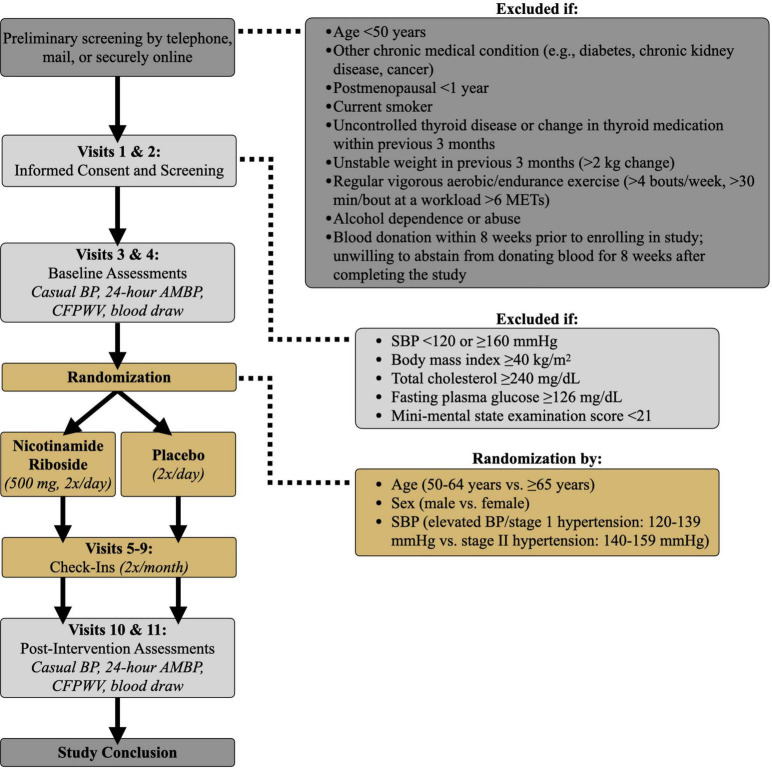
Study design. BP, blood pressure; AMBP, ambulatory blood pressure; SBP, systolic blood pressure; CFPWV, carotid-femoral pulse wave velocity; METs, metabolic equivalents.

Casual (resting) SBP and DBP will be measured and classified according to 2017 American College of Cardiology/American Heart Association guidelines (3). Subjects will rest quietly in the seated position for at least 5 min with their back supported, feet flat on the floor, and arm at heart level. BP will be measured in triplicate, with a 2-min recovery between each measure, over the brachial artery of the non-dominant arm *via* automated oscillometric sphygmomanometer (Mindray), validated according to the Association for the Advancement of Medical Instrumentation standards and regularly calibrated by the manufacturer (28). Casual SBP, DBP, and pulse pressure will be defined as the average of the 3 measurements. Casual BP will be measured on 2 separate days at least 24 h apart both at baseline and at the end of the 3-month treatment period (Figure 2) and averaged over the 2 measurement days. Casual BP also will be measured every 2 weeks during the supplementation period.

### Secondary Outcomes

#### Twenty-Four Hour Ambulatory Blood Pressure

Brachial artery BP will be measured every 20 min during the day (16-h period) and every 60 min at night (8-h period) over a 24-h period according to each participant’s individual sleep-wake cycle. BP will be measured *via* ambulatory BP monitoring (Oscar 2, SunTech Medical), validated according to British Hypertension Society standards (29). Participants will be outfitted with the ambulatory BP monitor by a trained investigator and given written and verbal instructions regarding monitor operation. The BP cuff will be placed securely over the non-dominant arm. Participants also will be given a diary to record physical activity or psychological events that occur over the 24-h period that may influence BP measurements. Ambulatory BP recordings will be analyzed for mean 24-h, daytime, and nighttime SBP, DBP, and pulse pressure.

#### Aortic Stiffness

Aortic stiffness will be determined *via* CFPWV, the gold standard, non-invasive technique for measurement of arterial stiffness in humans (30, 31). Pressure waveforms will be recorded using the Non-Invasive Hemodynamic Workstation (NIHem; Cardiovascular Engineering, Inc.) with simultaneous electrocardiograph (ECG) recording. Mean distance will be calculated as the distance between the suprasternal notch and femoral measurement site minus the distance between the suprasternal notch and carotid measurement site. Aortic transit time will be calculated as the time between the R-wave and foot of the femoral pressure wave minus the time between the R-wave and foot of the carotid pressure wave. CFPWV will be calculated as mean distance divided by transit time.

### Other Outcomes

#### Safety and Tolerability

All subjects will report to the laboratory every 2 weeks during the 3-month treatment period (Visits 5–9, Figure 2) to meet with a study nurse. Safety, i.e., risk to the subject, will be assessed by recording the number and severity of adverse events. Tolerability, i.e., the degree to which overt adverse events are tolerated by participants, will be determined based on the number of participants that drop out of the study due to adverse events.

#### Adherence

Subjects will be instructed to bring their pill containers to their bi-weekly check-in visits throughout the treatment period. The study nurse will count the number of pills remaining in the returned container and the percentage of prescribed pills consumed by participants will be used to assess adherence to the intervention.

#### Nicotinamide Adenine Dinucleotide^+^ Bioavailability

Assessment of the NAD^+^ metabolome (i.e., NAD^+^ and NAD^+^-related metabolites) will be performed using whole blood sampled before and after the 3-month intervention. EDTA-anticoagulated whole blood will be collected and mixed with a nucleotide internal standard containing uniformly labeled U13C yeast extract and heated deoxygenated buffered ethanol solution, as described previously (32). The sample will be microfuged at 13,200 g and 4°C and the supernatant stored at –80^°^C until analysis.

#### Mechanistic Biomarkers

Venous blood samples from the antecubital area will be obtained following 20 min of quiet rest. Blood samples will be analyzed by the Colorado Clinical Translational Sciences Institute core laboratory for plasma endothelin-1 (potent vasoconstrictor; ELISA; R&D Systems), plasma norepinephrine (marker of sympathoadrenal activity; high-performance liquid chromatography; BioRad Labs), plasma oxidized low-density lipoprotein (marker of oxidative stress; ELISA; Mercordia), plasma total antioxidant status (marker of antioxidant defenses; colorometric method; Randox Laboratories), interleukin-1β and interleukin-6 (inflammatory markers; ELISA; R&D Systems).

### Selection and Treatment of Subjects

#### Study Location and Timeline

All study visits will be performed at the Clinical Translational Research Center (CTRC) at the University of Colorado (CU) Boulder Campus. To account for an approximate 20% dropout rate, we plan to enroll 118 subjects to achieve our goal of completing testing on 94 participants.

#### Recruitment and Eligibility

Recruitment efforts will include newspaper, radio, and social media advertisements; CU Boulder campus email bulletins sent to faculty, staff, and students; and flyers handed out at senior centers and community fairs in the Boulder/Denver area. Interested individuals will contact the laboratory *via* telephone or email information provided in the recruitment materials and be directed to fill out a general screening form to determine their eligibility for the study. Potential participants who meet the initial inclusion/exclusion criteria will then be contacted and scheduled for consent and screening visits. Individuals who do not meet inclusion/exclusion criteria will also be notified. Individuals will be allowed to ask any questions they have about the study at any point in the pre-consent process. All email, phone, and/or mail communication with potential participants will be kept confidential.

#### Informed Consent and Screening

Informed consent will be obtained either in-person or electronically over Zoom and DocuSign platforms. A trained and approved member of the study team will give an overview of the study and all study procedures, and the participant will be given time to read through the informed consent document and invited to ask any questions. Both the participant and the investigator obtaining consent will sign the last page of the consent form and a copy of the signed consent form will be provided to the participant. All study procedures have been reviewed and approved by the Institutional Review Board (IRB) at CU Boulder (Protocol Number: 18-0644).

Following informed consent, participants will undergo screening assessments to further determine their eligibility for meeting enrollment criteria (Visits 1–2, Figure 2). Participants will be asked to abstain from non-prescribed over-the-counter medications and supplements for 48 h; food and caffeine for 12 h; and alcohol and strenuous exercise for 24 h. Consent and screening procedures will be performed within a 1-week period to determine if participants qualify for the study. Screening assessments will include:

• Resting BP and heart rate
• Height and body weight
• Medical history
• Questionnaires and paperwork
• Family medical history
• Physical activity *via* the Modifiable Activity Questionnaire (33, 34)
• Cognitive function *via* the Mini Mental State Exam (35)
• Screening blood collection.

Once all screening procedures have been performed, the participant’s results will be reviewed by the medical director on the research protocol. The medical director will approve of each individual’s eligibility for participation in the study. Subjects taking antihypertensive medications will be included provided they meet inclusion/exclusion criteria (Table 1). Medication regimen (prescription and dosing) must be stable for 3 months prior to enrollment and remain stable throughout the study.

#### Baseline Assessments

Eligible participants who qualify for the study based on previous screening visits will undergo baseline experimental testing within a 10-day period (Visits 3 and 4, Figure 2). Participants will be asked to avoid any major lifestyle changes for the duration of the study such as changes in physical activity, diet, and body weight, unless changes are deemed necessary by their physician. Subjects also will be asked to maintain a stable medication regimen throughout the study and notify the study team should their physician recommend any immediate changes in medication. Physical activity will be recorded before and after the intervention *via* accelerometer (36) (GENEActive; 3-day recording) and the Community Healthy Activities Model Program for Seniors questionnaire (37). Automated Self-Administered 24-h dietary recall (38) (ASA24; National Cancer Institute) also will be completed to ensure participant dietary composition remains unchanged after vs. before the intervention.

#### Randomization and Blinding

After baseline assessments, participants will be randomized to 3 months of nicotinamide riboside or placebo supplementation. Block randomization will be employed based on age (50–64 years vs. ≥ 65 years), sex (male vs. female), and SBP (elevated BP/stage 1 hypertension: 120–139 mmHg vs. stage II hypertension: 140–159 mmHg). Randomization will be performed by a staff research assistant who is not involved with data acquisition or analysis and will remain unblinded for the duration of the study. The Microsoft Excel random number generator function will be used to assign participants to nicotinamide riboside or placebo treatment. Investigators responsible for collection and analysis of outcome measurements will be blinded to participant group assignment. Participants also will be blinded to their treatment group.

#### Intervention

Participants assigned to the experimental treatment group will take 500 mg of nicotinamide riboside (NIAGEN^®^.; Chromadex, Inc.) twice per day (1,000 mg/day total) for 3 months. Each nicotinamide riboside capsule contains 250 mg of nicotinamide riboside chloride mixed with microcrystalline cellulose within a vegetarian capsule. Nicotinamide riboside is generally recognized as safe by the Federal Drug Administration (FDA; GRAS No. 635) and is being studied under an Investigational New Drug status (IND; No. 137994). Participants assigned to the placebo treatment group will take placebo pills twice a day for 3 months. Placebo pills contain microcrystalline cellulose within a vegetarian capsule. ChromaDex, Inc. will supply both nicotinamide riboside and placebo pills and ship them to the CU Boulder CTRC. Bottles containing 2-week supplies of nicotinamide riboside or placebo will be prepared by the CU Boulder CTRC pharmacy and labeled with the participant’s name and dosing instructions. The bottles will be distributed to each participant every 2 weeks by a study investigator who will provide verbal instructions for pill dosing and timing.

During the intervention period, participants will come to the CU Boulder CTRC every 2 weeks to provide a urine sample; measure BP, heart rate, and body weight; report any symptoms to a CTRC nurse; notify the study team of any medication changes; return unused pills; and receive their next 2-week pill supply (Visits 5–9, Figure 2).

#### Post-intervention Assessments

After completing 3 months of either nicotinamide riboside or placebo treatment, participants will return to the CU Boulder CTRC for reassessment of all outcome measures within a 10-day period (Visits 10 and 11, Figure 2). Participants will continue to take their assigned pills throughout post-testing to maintain the treatment stimulus. However, on testing days, participants will not take nicotinamide riboside for at least 12 h prior to their visit and will wait to take their pills until after their visit ends to avoid any acute effects of the dose on our outcome measures. All post-assessment measurements will be identical to, and made under the same experimental conditions as, assessments performed at baseline.

#### Study Withdrawal

Participants can withdraw their informed consent at any time and for any reason. Investigators may also remove participants from the study at any time due to significant non-compliance with the protocol (i.e., procedures, assessments); any adverse event that, in the opinion of the medical director, indicates that continuing in the study is not in the best interest of the participant; loss of the participant’s ability to freely provide consent; or other serious changes in physical or mental health.

#### Adverse Events

Participants will be instructed to inform members of the study team or CTRC staff of any side effects or adverse events. Mild and moderate adverse events will be regularly reported to the IRB, NIH, and FDA. Serious adverse events will be reported to the CU Boulder IRB within 24 h of occurrence. An unanticipated adverse event which meets the CU Boulder IRB definition of an unanticipated problem (i.e., any unanticipated and undesirable effect arising from participation in research that results in an increased risk of harm or injury to a subject or which suggests the possibility of increased risks to other subjects) will be reported to the CU Boulder IRB using the corresponding form within 5 days of occurrence.

#### Statistical Design, Power, and Analysis Plan

Sample size calculations and pre-determined statistical power for this randomized, double-blind, placebo-controlled, parallel group design clinical trial are based on the primary outcome, casual SBP. Sample size and power were estimated based on preliminary data from our crossover design pilot trial on nicotinamide riboside supplementation in midlife and older adults (22). In subjects from our pilot trial who had initial above-normal SBP, the change from baseline in SBP with nicotinamide riboside supplementation was –12.8 mmHg (i.e., SBP decreased) and –4.2 mmHg with placebo treatment, resulting in a difference of 8.6 mmHg between treatment arms. The standard deviation of change was estimated as 11.6 mmHg for the nicotinamide riboside condition and 13.5 mmHg for the placebo condition. This yielded an estimated effect size of 0.68, which was used to determine the required sample size for the current protocol. It was determined that 47 subjects per group (94 total) would be required to achieve a power of 90% with a two-sided significance level of α = 0.05. To account for a potential drop-out rate of 20%, i.e., the rate observed in our pilot study, a total of 118 participants will be enrolled and equally randomized to an intervention group, with 59 participants/group.

Descriptive statistics will be provided for all baseline and outcome variables. Means and standard deviations will be calculated for all continuous variables, proportions will be calculated for categorical or ordinal variables, and 95% confidence intervals will be provided when appropriate. We will use the *t*-test to compare the change in outcome measures between nicotinamide riboside and placebo treatment groups because all outcome variables are continuous. All outcome variables will be assessed and confirmed for normality and data transformations will be performed, if necessary, prior to performing comparisons. All comparisons will be made on intention to treat principle and conclusions will be made using a two-sided significance level of α = 0.05. Furthermore, linear regression will be used to adjust for potential confounders such as age, baseline BP, and use of antihypertensive medications. We are aware that statistical significance does not imply clinical significance; thus, we will make clinical interpretations carefully.

#### Data Management

All participant identities and records will be kept strictly confidential; only the principal investigator and research staff will have access to data from this study. Physical data will be stored in locking file cabinets in a secured-entry laboratory space. Electronic data will be stored on a laboratory server, which is accessed by individual user sign-on and passwords. The names of subjects will not be identified in any publication arising from this study. If individual participant data is presented in presentations or manuscripts, it will not be associated with any information that could be used to identify participants in the study.

#### Data Safety Monitoring

This research protocol is subject to oversight by an internal CTRC safety monitoring committee and an external safety officer, who is completely independent of the study team. Additionally, a physician investigator also will provide day-to-day medical oversight to ensure participant safety by serving as medical director on this protocol. The study team will be in regular contact with the medical director and review enrollment and safety data with the safety officer at least twice per year.

## Expected Results

Based on the results from our previously published pilot study (22), we expect the following effects of 3 months of nicotinamide riboside vs. placebo supplementation in our proposed study population of adults aged 50 years and older with above-normal SBP (120–159 mmHg) at baseline.

Primary Outcome: We expect casual SBP to decrease.

Secondary Outcomes: We expect 24-h ambulatory SBP to decrease as a result of decreases in both daytime and nighttime SBP. We expect CFPWV (aortic stiffness) to decrease.

Other Outcomes: We expect nicotinamide riboside treatment to be safe, well-tolerated, and associated with high adherence. We expect casual and 24-h ambulatory DBP and pulse pressure will decrease. We expect concentrations of NAD^+^, NAD^+^-related metabolites, and total antioxidant status to increase and concentrations of endothelin-1, norepinephrine, oxidized low-density lipoprotein, interleukin-1β and interleukin-6 to decrease in response to nicotinamide riboside treatment.

## Discussion

The age-related increase in SBP serves as a key modifiable risk factor linking advancing age to chronic conditions such as CVD, chronic kidney disease, and cognitive decline (1, 3). Given that roughly two-thirds of adults 50 years and older have above-normal BP, this population is at high risk for disease development (1). Moreover, the number of midlife and older adults is projected to rise exponentially in the coming years (39). Together, these events create a biomedical need to investigate and identify novel strategies to prevent or slow declines in CV health to reduce the impending increase in chronic disease burden in the aging population (40).

Declines in intracellular NAD^+^ concentrations occur with advancing age and are associated with age-related chronic diseases (12, 14, 15). This may be due both to reductions in the synthesis of NAD^+^ as well as increases in the activity of NAD^+^-consuming enzymes, such as polyADP-ribose polymerases (PARPs), cyclic ADP-ribose synthases (e.g., CD38), and the mammalian family of sirtuins, which all affect key components of cellular homeostasis such as genome stability, mitochondrial function, and inflammation (11, 14, 41, 42). As such, compounds that increase NAD^+^ bioavailability may be viable therapies to improve physiological function with advancing age (19, 43). Indeed, administration of NAD^+^ precursors can effectively restore NAD^+^ bioavailability and improve physiological function in preclinical models of aging and age-related diseases (15, 21, 44). In midlife and older adults, supplementation with nicotinamide riboside may boost NAD^+^ bioavailability back toward young, healthy levels such that appropriate concentrations are available for enzymes involved in DNA repair, immune regulation, energy metabolism, and overall cellular maintenance (42).

Our initial crossover-designed pilot study was the first to report elevations in NAD^+^ concentrations after chronic supplementation of nicotinamide riboside in humans (22). In addition, the increase in NAD^+^ concentrations was associated with clinically significant reductions in SBP and arterial stiffness (22, 24, 45). Based on these encouraging results, we hypothesize that nicotinamide riboside treatment also may hold promise for improving CV function in humans, specifically reducing SBP and arterial stiffness, which may reduce the risk for developing age-related chronic diseases. Moreover, as available antihypertensive pharmacotherapies do not impact NAD^+^ bioavailability, supplementation with nicotinamide riboside may provide additional benefits for CV aging beyond prescription medications.

We have elected to use nicotinamide riboside based on the promising results of our pilot clinical trial; however, additional NAD^+^ precursors also are available for human research, such as nicotinamide mononucleotide and nicotinamide. Nicotinamide mononucleotide and nicotinamide are currently under investigation in healthy and patient populations for their potential beneficial effects on cardiac function, surgery-related acute kidney injury, glucose tolerance, insulin sensitivity, and cardiorespiratory fitness (17). However, the effects of NAD^+^ precursors other than nicotinamide riboside on vascular health remain to be determined in humans. Niacin (i.e., vitamin B_3_ or nicotinic acid) is a more thoroughly researched NAD^+^ precursor that may have a limited effect for reducing CV events or mortality (46, 47). However, niacin induces undesirable side effects (primarily “flushing”) at therapeutic doses, precluding it from regular clinical use. Moreover, niacin may not increase NAD^+^ concentrations as potently as nicotinamide riboside (48). Thus, given the initial safety, tolerability and efficacy of nicotinamide riboside for augmenting NAD^+^ concentrations and improving CV function (22), this specific compound warrants additional investigation.

This study will assess the safety and efficacy of 3 months of dietary supplementation with nicotinamide riboside for improving CV function in midlife and older adults with above-normal SBP at baseline. Our previous pilot study performed a majority of the procedures outlined in the current protocol and utilized the same intervention compound (22), whereas 24-h SBP, a key secondary outcome of the current trial, has been successfully assessed previously by our laboratory (49). Thus, we do not expect any significant problems in conducting this protocol. Should the hypothesized effects of the intervention occur, additional analyses beyond those outlined herein also could be performed to further assess potential mechanisms of action, such as reductions in chronic inflammation or endothelial activation.

Through the completion of this study, we will determine whether 3 months of nicotinamide riboside treatment: (1) lowers casual BP, 24-h BP, and aortic stiffness; and (2) is safe, tolerable, and associated with good adherence. We also will gain insight into the potential mechanisms that may mediate nicotinamide riboside-related improvements in CV function. This protocol serves as an essential next step in the translation of NAD^+^-boosting compounds for CV health. If our hypotheses and expected results are confirmed, data from this phase IIa clinical trial would provide the foundation for a multi-site phase III clinical trial to assess the efficacy of dietary supplementation with nicotinamide riboside in an even larger cohort while enabling further assessment of the compound’s safety profile through monitoring of adverse events. Moreover, results from this trial also may inform future clinical trials to extend any observed CV benefits of dietary supplementation with nicotinamide riboside to other clinical populations.

## Ethics Statement

Thestudies involving human participants were reviewed and approved by the University of Colorado, Boulder, IRB. The patients/participants provided their written informed consent to participate in this study.

## Author Contributions

CM, DS, and DC conceived of the overall study design. ZY assisted with the design of statistical analyses. MC provided medical oversight on the study protocol. KF, DC, and DS drafted the manuscript. All authors refined and approved of the final manuscript.

## Conflict of Interest

The authors have a material transfer agreement with the ChromaDex External Research Program (CERP) to obtain the study compound. The authors declare that the research was conducted in the absence of any commercial or financial relationships that could be construed as a potential conflict of interest.

## Publisher’s Note

All claims expressed in this article are solely those of the authors and do not necessarily represent those of their affiliated organizations, or those of the publisher, the editors and the reviewers. Any product that may be evaluated in this article, or claim that may be made by its manufacturer, is not guaranteed or endorsed by the publisher.
